# Book Review

**Published:** 1920-12

**Authors:** 


### Reviews of Books.

**Bibliography of William Henry Welch, M.D., LL.D.** Prepared by WALTER C. BURKET, M.D. Pp. 47. Baltimore: The Johns Hopkins Press. 1917.—As a tribute to the scholarship and public services of Dr. Welch of the Johns Hopkins University this bibliography of his writings has been published separately, so that all may know where the writings were originally published and their chronological order. The wide range of topics covered in these writings conveys faintly but unmistakably the learning and industry of their distinguished author.

**Child Welfare.** By J. SIM WALLACE, D.Sc., M.D., L.D.S. Pp. x., 102. London: Baillière, Tindall & Cox. 1919. Price 5s. net.—A most interesting book and well worth reading by all interested in this most important subject. The importance of the saliva for oral hygiene and prevention of dental caries is well indicated, as well as the dietary that would leave the mouth in a state of physiological cleanliness after meals. No allusion is made to the possible action, as an antiseptic, of the sulphocyanide of potassium in the saliva. Dental caries and diseases of the mouth would be enormously reduced if the instructions in the book were carried out.

**Practical Guide to the Diseases of the Throat, Nose, and Ear.** By WM. LAMB, M.D., C.M. Edin. Pp. xi., 372. London: Baillière, Tindall & Cox. Price 8s. 6d. net.—The author has set out to provide in some 350 pages a guide to meet the needs of senior students and junior practitioners. The difficulties facing the author of such a work is to know how much to include and the success of the present volume may be gauged by the fact that it is the fourth edition which is now under review. It will be found eminently suitable to students while undergoing practical instruction in these special departments. The author has perhaps erred in the direction of a too detailed description of some operations, thus sacrificing space which could have been more usefully employed for the detail of signs and symptoms.

The operation as described for removal of the tonsils is rather difficult to follow, but as it is impossible to become proficient in this operation without considerable practice this is not of great importance. The formulæ at the end are well selected, and will probably be found one of the most valuable portions of a generally excellent work.

**Diseases of the Ear in School Children.** By JAMES KERR LOVE, M.D. Pp. viii., 94. Bristol: John Wright & Sons Ltd. Price 5s. 6d. net.—The seeds of deafness in the adult are sown in childhood, and progress in the prevention of deafness in the future can only be obtained by attention to the child. The author's object in producing this work has been to emphasise this cardinal fact, and to show what excellent results can be produced by the thorough and skilled treatment of aural disease in childhood. He has been in the fortunate if not unique position of having charge of a large group of clinics in Glasgow devoted entirely to the aural treatment of cases drawn from some 130,000 school children. Provision is made for continuous treatment, where necessary, by a staff of nurses, but where operative treatment is necessary the cases are referred to the nearest general or special hospital. As a result of this experience he has come to the very important conclusion that "chronic middle-ear suppuration is during the school period a disease which is very amenable to treatment if the latter be conducted continuously by a competent nurse." The effects of syphilis and hereditary deafness are fully considered.

No one reading this work can fail to be convinced of the essential importance of the specialist treatment of ear diseases in school children. It should be carefully read by all those who have any responsibility for the medical care of school children, and if as a result other centres be induced to follow the lead of Glasgow the author will have earned a debt of gratitude from future generations.

**Orthopædic Effects of Gunshot Wounds.** By S. W. DAW. Pp. xi., 242. London: Henry Frowde, Hodder & Stoughton. Price 7s. 6d. net.—This little book of the Oxford War Series is of the utmost value, and is published at a most opportune moment. It presents the reader with a concise summary of the principles and practice of orthopædic surgery as evolved at the leading Special Military Surgical Hospitals. Now that such a large proportion of medical men are in constant contact with disabled soldiers, it is incumbent on all to discover the means which have proved to be the most efficient in restoring wounded men and refitting them for the ordinary duties of civil life. The introductory notes are especially valuable, and should be impressed on every Medical Officer working in the Ministry of Pensions. They are the Ten Commandments of the orthopædic surgeon, and the greatest of these is established in the words, "Aim at restoring function, not anatomical structure."

The author takes us through the various common injuries of the upper limb, trunk, and lower limb. This is followed by the consideration of nerve lesions. This section might be improved by better diagrams. Finally, we have a most valuable chapter on "Functional Disabilities" by Dr. Cuthbert Morton.

While the book as a whole is extremely valuable, it is impossible not to feel a certain amount of regret that other well-known and proved methods of treatment have not been referred to. Verrall's excellent splints for pronation and supination and his finger flexion splint deserve a place in this work. A few paragraphs might with advantage have been devoted to the details of modern plaster of Paris work and to the preparation of celluloid splints. The book is well written, and deserves wide circulation amongst both advanced students and practitioners.

**Shell-shock and its Lessons.** By G. Elliott Smith, M.D., F.R.S., and T. H. Pear, M.A., B.Sc. Pp. xv., 135. Manchester: The University Press. 1919. Cheap Edition. Price 1s. 6d. net.—This is a cheap impression of the second edition of a most interesting book, which was first published in 1917. The authors are the Professor of Anatomy and the Lecturer in Experimental Psychology in the University of Manchester. The whole volume presents so compact and well-ordered a discussion of the subject that it is difficult to detect two minds and two pens at work. It is not, as the title might imply, merely devoted to the symptomatology and treatment of soldiers who in the recent war proved unequal to the mental stress and strain of battle. The interest it evokes is wider and longer lasting. Using as a text the now familiar phenomena of the "War Neuroses," the authors plead temperately but very forcibly for more enlightened methods of dealing with the early manifestations of psychical disturbance. Their conclusion illustrates the object with which the book has been written: "It is our bounden duty as a nation to take measures such as most civilised countries have adopted some time ago. . . . There should be hospitals to which patients in the early stages of mental disturbance can go, without any legal formalities, and receive proper treatment from physicians competent to diagnose their troubles and to give them appropriate advice." We can only add that it is a consummation devoutly to be wished.

**Map Showing Distribution in England and Wales of the Anopheline Mosquitoes.** By William D. Lang, M.A. Pp. 63. London: British Museum (Nat. Hist.). 1918. Price 2s. 6d. net.—This pamphlet deals with the present state of our knowledge regarding the distribution of the three species of anopheles which have been found in England and Wales. The three species are *A. Maculipennis*, *A. Bifurcatus*, and *A. Plumbeus*, of which the first two are proved malaria carriers. The map is valuable in that it gives an easy means of reference, but as the compiler points out it shows chiefly where anopheles have been most sought for. There are many blank places on the map which should stimulate collectors to fill them up. The pamphlet includes clear information how to distinguish anopheles from other insects and the one species of anopheles from another and what are their favourite breeding places.

It would have been interesting to have marked on the map places where malarial infection has been actually transmitted in the last four years by returned soldiers to persons who have not been abroad. It is also important to know the optimum temperature for the development of malaria in these British species of anopheles, so that we might know during which months there is a possibility of fresh cases arising. Anyone who wishes to add fresh rings to this map should have no difficulty in doing so, as the Bristol area does not seem to have received much attention.

**The New Physiology.** By J. S. Haldane, M.D., LL.D., F.R.S., Pp. vii., 156. London: Charles Griffin & Co. Ltd. 1919.—This is a volume of addresses, the third of which gives its title to the collection. Running through them all, and variously illustrated by Dr. Haldane's great knowledge and practical physiological experience, is his well-known metaphysical conception of the place of biology in relation to the other sciences. From this point of view the last essay, "Are Physical, Biological and Psychological Categories Irreducible?" is perhaps the most interesting. Its concluding paragraph sums up, as far as it is possible to do so, the author's position: "From the point of view of each individual science, there is a conflict of categories or fundamental hypotheses with those of other sciences; but from the wider standpoint of philosophy these categories are only provisional working hypotheses. . . . We must regard categories as only forms which the riches of this spiritual world pass through in the course of their ever fuller manifestation."

The peculiar value of Dr. Haldane's contributions to the philosophy of living matter lies of course in his personal researches into physiological processes. His method is not to pick out such recorded experiments and observations of others as may support his own philosophical position, but rather from his own experimental results to construct an accordant philosophy.

To medical men the address on the relation of physiology to medicine will naturally be of especial interest. Here, too, the author's terse and pithy style is perhaps at its best. Discussing the teaching of anatomy, he remarks: "Human anatomy behaves as if she had sold her scientific birthright for a sorry mess of 'systematic' pottage." Again, on pathology: "To many medical men the pathologist is a sinister figure which stalks behind the chaplain and in front of the undertaker." And on practical medicine: "It is 'with brains, sir,' that the physician mixes his prescriptions and the surgeon guides his knife."

Dr. Haldane strongly supports the establishment of clinical laboratories under a trained and paid assistant, to carry out research work under the direction of the physicians and surgeons of hospital staffs. The assistant should be in touch with the main University laboratories, and able to obtain assistance and information from this source. He also is in favour of whole-time professorships of medicine and surgery.

It is impossible in a short review to do justice to the varied interests of Dr. Haldane's book, but we trust that we have said enough to stimulate all who take their profession seriously to get the book and read it, not once nor twice; to work out for themselves the many trains of thought which it suggests, and to consider carefully and critically the fundamental principles on which it is based.

J. M. F.-B.

**The Sympathetic Nervous System in Disease.** By W. Langdon Brown, M.A., M.D. Cantab., F.R.C.P. Lond. Henry Froude, Oxford Univ. Press. London: Hodder & Stoughton Ltd., Warwick Square, E.C. Price 10s. 6d. net.—This work is an embellished reproduction of the Croonian Lectures delivered by Dr. Langdon Brown in 1918, and deals especially with the influence of the sympathetic in such conditions as diabetes insipidus, glycosuria, disordered gastric digestion, and diseases of the circulatory system.

As an introduction the author has succeeded in giving a very lucid anatomical and physiological description of the sympathetic and parasympathetic systems, containing much of clinical interest from both a medical and surgical point of view.

The views expressed concerning the functions of the endocrine glands are based on the fact that the adrenals, the thyroid, and the pituitary are each stimulated by the sympathetic, and are all capable of lowering the carbohydrate tolerance; and the whole question of sugar metabolism receives very full and careful treatment, while the importance of exact clinical observations in reference to hyperglycæmia and glycosuria is illustrated by many interesting cases observed by the author.

We would particularly commend to the notice of surgeons Chapter iv., with its explanations of symptoms occurring in cases of duodenal ulcer with pancreatic adhesions, appendicitis, and of atonic dilatation of the stomach with hyperchlorhydria.

Although attributing enormous importance to the functions of the sympathetic, Dr. Langdon Brown does not hesitate to dethrone the vasomotor system, and therefore the sympathetic, from the autocratic control of the circulation hitherto assigned to it in physiology and pathology; and in referring to shock, he maintains that this condition occurs not through paralysis of but in spite of the action of the vasomotor system.

